# The Role of *hOGG1* C1245G Polymorphism in the Susceptibility to Lupus Nephritis and Modulation of the Plasma 8-OHdG in Patients with Systemic Lupus Erythematosus †

**DOI:** 10.3390/ijms16023757

**Published:** 2015-02-09

**Authors:** Hui-Ting Lee, Chen-Sung Lin, Chyou-Shen Lee, Chang-Youh Tsai, Yau-Huei Wei

**Affiliations:** 1 Institute of Clinical Medicine, National Yang-Ming University, Taipei 112, Taiwan; E-Mails: htlee1228@gmail.com (H.-T.L.); doc2765c@ms59.hinet.net (C.-S.L.); 2 Faculty of Medicine, National Yang-Ming University, Taipei 112, Taiwan; 3 Department of Medicine, Mackay Medical College, New Taipei City 252, Taiwan; E-Mail: amy@mmh.org.tw; 4 Division of Allergy, Immunology and Rheumatology, Department of Internal Medicine, Mackay Memorial Hospital, Taipei 104, Taiwan; 5 Division of Thoracic Surgery, Taipei Hospital, Ministry of Health and Welfare, New Taipei City 242, Taiwan; 6 Mackay Junior College of Medicine, Nursing, and Management, New Taipei City 252, Taiwan; 7 Division of Allergy, Immunology and Rheumatology, Department of Medicine, Taipei Veterans General Hospital, Taipei 112, Taiwan; 8 Institute of Biochemistry and Molecular Biology, National Yang-Ming University, Taipei 112, Taiwan

**Keywords:** 8-hydroxy-2'-deoxyguanosine (8-OHdG), human 8-oxoguanine glycosylase 1 (*hOGG1*) C1245G polymorphism, lupus nephritis, systemic lupus erythematosus (SLE)

## Abstract

We investigated whether the C1245G polymorphism of human 8-oxoguanine glycosylase 1 (*hOGG1*) gene confers the susceptibility to systemic lupus erythematosus (SLE) occurrence of lupus nephritis and affects the plasma level of 8-hydroxy-2'-deoxyguanosine (8-OHdG) in patients with SLE. A total of 45 healthy controls and 85 SLE patients were recruited. The C1245G polymorphism of the *hOGG1* gene was determined by direct sequencing. The frequency of occurrence of the *hOGG1* 1245 GG genotype in SLE patients was 31.8% (27/85), which is lower than that of healthy controls of 53.3% (24/45). Thirty-three (33/85, 38.8%) SLE patients developed lupus nephritis. Significantly, SLE patients harboring the *hOGG1* 1245 GG genotype had a higher incidence to develop lupus nephritis than did those harboring the *hOGG1* 1245 CC or CG genotype (15/27, 55.6% *vs.*18/58, 31.0%, *p* = 0.031). Divided into subgroups, SLE patients harboring the *hOGG1* 1245 GG genotype had the highest plasma levels of 8-OHdG among patients with all genotypes, with regard to the coexistence of lupus nephritis (*p* = 0.020, ANOVA), including those with nephritis harboring the *hOGG1* 1245 CC or CG genotypes (*p* = 0.037), those without nephritis harboring the *hOGG1* 1245 GG genotype (*p* = 0.050), and those without nephritis harboring the *hOGG1* 1245 CC or CG genotype (*p* = 0.054). We conclude that the C1245G polymorphism of *hOGG1* may be one of the factors that confer the susceptibility to lupus nephritis and modulate the plasma level of 8-OHdG in patients with SLE.

## 1. Introduction

Systemic lupus erythematosus (SLE) is a systemic autoimmune disease characterized by the generation of arrays of pathogenic auto-antibodies that cause cascades of organ damages [1]. Similar to several human degenerative diseases, elevated reactive oxygen species (ROS) and ROS-triggered oxidative damages have been demonstrated to be involved in the pathogenesis of SLE [2,3,4,5]. Theoretically, ROS can attack the intracellular molecules randomly, e.g., protein, lipid, RNA and DNA, and thereby cause cellular dysfunction. Among these oxidative DNA damages, the formation of 8-hydroxy-2'-deoxyguanosine (8-OHdG), an oxidative damage product of 2'-deoxyguanine (dG), is the most common one [6]. Normally, the dG is matched with 2'-deoxycytosine (dC) during DNA replication. However, if the 8-OHdG is not repaired efficiently and accumulated in the affected tissues, it would pair wrongly with 2'-deoxyadenine (dA) rather than dC, and cause a G→C to T→A transversion [7]. As a result, if the affected gene harbors 8-OHdG, functional impairment may subsequently develop.

In human cells, 8-OHdG is mainly repaired by human 8-oxoguanine glycosylase 1 (*hOGG1*) through the base excision repair mechanism [8]. There have been three *hOGG1* genotypes identified because of a C to G shifting at base-pair (bp) 1245 (C1245G) in exon 7 of the *hOGG1* gene. Such a shifting causes a serine (Ser) to cysteine (Cys) substitution at codon 326 during translation. This phenomenon is referred to as *hOGG1* C1245G polymorphism. Considering the DNA repair efficiency, homozygous *hOGG1* 1245 CC genotype has the highest DNA repair activity followed by the heterozygous *hOGG1* 1245 CG genotype and then the homozygous *hOGG1* 1245 GG genotype in order [9]. This discrepancy in *hOGG1* repair activity implies that *hOGG1* 1245 GG genotype with lower efficiency in DNA repair might be related to a higher level of 8-OHdG in human tissues [10], and may contribute to the development of several human degenerative diseases, including type 2 diabetes mellitus [11], Huntington’s disease [12], chronic obstructive pulmonary disease [13], Graves’ ophthalmopathy [14], and higher susceptibility to cancer formation, including lung cancer [15] and esophageal squamous cell carcinoma [16]. However, the role of *hOGG1* C1245G polymorphism in the pathogenesis of SLE has remained obscure.

Lupus nephritis is a serious clinical manifestation of SLE. Studies revealed that excessive ROS production and oxidative stress may exacerbate inflammation, dampen antioxidant capacity and lead to tissue damage and the formation of lupus nephritis in SLE patients [3,17]. Furthermore, some authors also reported that *hOGG1* 1245 GG genotype is related to higher 8-OHdG content in patients with end-stage renal disease (ESRD) [18,19]. In one of our previous studies, we demonstrated that SLE patients had higher plasma levels of 8-OHdG than did healthy controls [20]. In light of these findings, we aimed to appraise (1) whether the *hOGG1* 1245 GG genotype contributes to a higher rate of SLE development and higher plasma level of 8-OHdG; (2) whether the *hOGG1* 1245 GG genotype confers a higher incidence to the development of nephritis in SLE patients; and (3) whether the *hOGG1* 1245 GG genotype modulates the plasma level of 8-OHdG in SLE patients with or without nephritis.

## 2. Results and Discussion

### 2.1. Distributions of hOGG1 C1245G Polymorphism in Healthy Controls and SLE Patients

**Figure 1:**
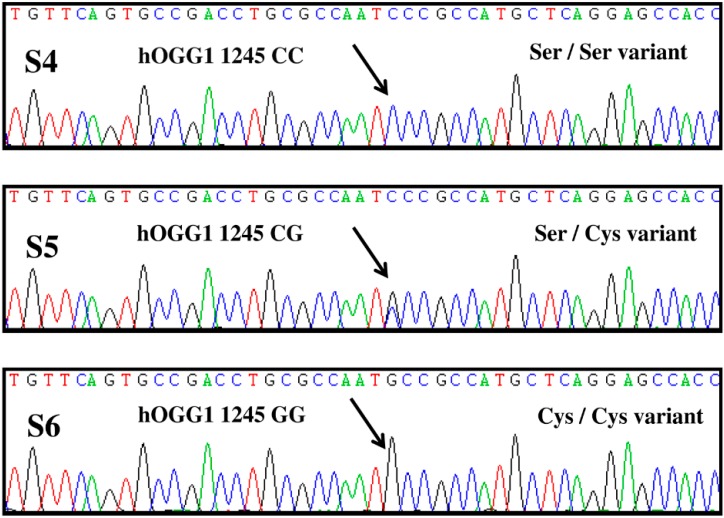
Illustrations are the results of direct sequencing of the DNA segment harboring the *hOGG1* gene. Green, red, blue and black colors represent A, T, C and G, respectively. systemic lupus erythematosus (SLE) patient S4 harbored a single C peak at bp 1245 (homozygous C/C allele) and belonged to the homozygous CC genotype. SLE patient S6 harbored a single peak of G at bp 1245 (homozygous G/G allele) and belonged to the homozygous human 8-oxoguanine glycosylase 1 (*hOGG1*) GG genotype. SLE patient S5 harbored concurrent C and G peaks at bp1245 (heterozygous C/G allele) and thus belonged to the heterozygous CG genotype.

The genotypes of *hOGG1* C1245G polymorphisms are illustrated in Figure 1. As revealed by direct sequencing, among the 45 healthy controls, 3 (6.7%) had the CC, 18 (40.0%) had the CG and 24 (53.3%) had the GG genotype, respectively. On the contrary, among the 85 SLE patients, 14 (16.5%) had the CC genotype, 44 (51.8%) had the CG genotype and 27 (31.8%) had the GG genotype, respectively. For the subsequent comparative analysis, CC genotype plus CG genotype were gathered as one group in this study (Table 1).

As shown in Table 1, among the 85 SLE patients, 27 (31.8%) harbored the GG genotype, and the rate was significantly lower than the 24 (53.3%) of the 45 healthy controls (*p* = 0.017). For further comparisons, healthy controls harboring the CC or CG genotypes were designated as Group A, healthy controls harboring the GG genotype were designated as Group B, SLE patients harboring the CC or CG genotypes were designated as Group C and SLE patients harboring the GG genotype were designated as Group D, respectively.

### 2.2. Levels of Plasma 8-OHdG in Healthy Controls and SLE Patients Based on the hOGG1 Gene Polymorphisms

**Figure 2:**
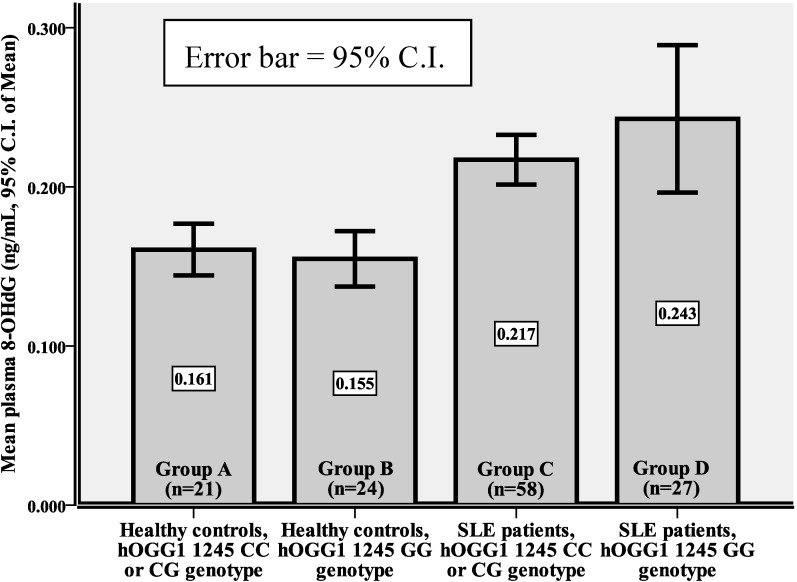
The plasma 8-OHdG in healthy controls and SLE patients with different *hOGG1* gene polymorphisms (mean levels and 95% C.I.).

As we reported previously, the plasma 8-OHdG levels (M ± SD) of the 45 healthy controls and 85 SLE patients were 0.157 ± 0.038 and 0.225 ± 0.082 ng/mL, respectively [20]. SLE patients had significantly higher plasma 8-OHdG levels as compared with healthy controls (*p <* 0.001), regardless of their *hOGG1* gene polymorphism of CC/CG genotypes (Group C *vs.* Group A, 0.217 ± 0.059 *vs.* 0.161 ± 0.036 ng/mL, *p <* 0.001) or GG genotype (Group D *vs.* Group B, 0.243 ± 0.117 *vs.* 0.155 ± 0.041 ng/mL, *p* = 0.001) (Table 2). Those harboring *hOGG1* 1245 GG genotype did not have a higher level of plasma 8-OHdG than those harboring CC or CG genotypes in healthy controls (Group B *vs.* Group A, 0.155 ± 0.041 *vs.* 0.161 ± 0.036 ng/mL, *p* = 0.614). A mild elevation of plasma 8-OHdG levels was observed in SLE patients with GG genotype (Group D *vs.* Group C, 0.243 ± 0.117 *vs.* 0.217 ± 0.059 ng/mL, *p* = 0.289), although it was not obvious. Among all subjects, Group D (patients with the *hOGG1* 1245 GG genotypes) had the highest plasma levels of 8-OHdG (*p <* 0.001, ANOVA, Table 2 and Figure 2).

**Table 1:** Distribution of *hOGG1* gene polymorphisms in healthy controls and SLE patients.

Subjects (Case Number, %)	*hOGG1* C1245G Polymorphisms		*p*-Value
	*hOGG1* 1245 CC or CG Genotype	*hOGG1* GG Genotype	
Healthy controls (*n* = 45, 100)	21 (46.7)—Group A	24 (53.3)—Group B	0.017 *
SLE patients (*n* = 85, 100)	58 (68.2)—Group C	27 (31.8)—Group D	

Group A: Healthy controls harboring the *hOGG1* 1245 CC or CG genotypes; Group B: Healthy controls harboring the *hOGG1* 1245 GG genotype; Group C: SLE patients harboring the *hOGG1* 1245 CC or CG genotype; Group D: SLE patients harboring the *hOGG1* 1245 GG genotype, * Comparison was made between healthy controls and SLE patients with regard to GG genotype.

**Table 2:** Levels of plasma 8-hydroxy-2'-deoxyguanosine (8-OHdG) in healthy controls and SLE patients as related to the *hOGG1* gene polymorphisms.

	Plasma 8-OhdG (M ± SD, ng/mL)		Plasma 8-OHdG (M ± SD, ng/mL)	*p*-Value
Healthy controls (*n* = 45)	0.157 ± 0.038	SLE patients (*n* = 85)	0.225 ± 0.082	<0.001 [20]
*hOGG1* C1245G polymorphisms		*hOGG1* C1245G polymorphisms		
**Group A** (CC or CG genotypes, *n* = 21)	0.161 ± 0.036	**Group C** (CC or CG genotypes, *n* = 58)	0.217 ± 0.059	<0.001 *
**Group B** (GG genotype, *n* = 24)	0.155 ± 0.041	**Group D** (GG genotype, *n* = 27)	0.243 ± 0.117	0.001 *
*p*-value	0.614 **	*p*-value	0.289 **	<0.001 ***

M, mean, SD, standard deviation; * compared between healthy controls harboring the CC or CG genotypes (Group A) and SLE patients harboring the CC or CG genotypes (Group C), and between healthy controls harboring the GG genotype (Group B) and SLE patients harboring the GG genotype (Group D); ** compared between healthy controls harboring the GG genotype (Group B) and those harboring the CC or CG genotypes (Group A), or between SLE patients harboring the GG genotype (Group D) and those harboring CC or CG genotypes (Group C); *** compared among Groups A, B, C and D (ANOVA).

We have demonstrated that SLE patients had a higher plasma level of 8-OHdG than did the healthy controls [20]. Since 8-OHdG is repaired by hOGG1 in human tissues, the possible influence of *hOGG1* 1245 GG genotype on SLE susceptibility or the elevation of 8-OHdG level in SLE patients deserves evaluation. In our study cohort, the percentage of *hOGG1* 1245 GG genotype in SLE patients was 31.8%, which was lower (*p* = 0.017) than that in healthy controls (24/45, 53.3%). On the contrary, the percentage of *hOGG1* CC genotype in healthy control was 6.7% (3/45), which was lower than in SLE patients (16.5%, 14/85). As reported in the published data, *hOGG1* 1245 GG genotype frequency in SLE patients is variable, higher in normal Taiwanese (20.5%) population [10] and Japanese populations (13.7%) [21], similar to normal Chinese population (31.4%) [18], and lower in another normal Taiwanese cohorts (37.6% [22] and 42.0% [19]). There is actually a wide range of frequency in the occurrence of *hOGG1* 1245 GG genotype in the normal Taiwanese population. Furthermore, the percentage of CC genotype in our healthy cohort was 6.7%, which was lower than those reported previously [10,18,19,21,22]. This might suggest that our healthy cohort had a lower repair activity for oxidative damage to DNA, but it was not that low to lead to a significant elevation of plasma levels of 8-OHdG compared with those found in SLE patients. Our results have indicated that *hOGG1* 1245 GG genotype does not predispose to SLE and is not the prominent contributor to a higher plasma 8-OHdG levels in SLE patients. However, if SLE patients harbor the *hOGG1* 1245 GG genotype (Group D), they might exhibit diminished capacity to ameliorate oxidative stress and resulted in higher plasma levels of 8-OHdG (Table 2 and Figure 2). Alternatively, there might be other contributing factors not yet found that also resulted in higher 8-OHdG. Although the CC genotype was previously reported as a wild type, it was mainly found in the European/American populations. The distribution variations of the *hOGG1* gene polymorphisms might be dependent on ethnic group. Thus, the CC, CG or GG genotype cannot be regarded as the wild type of the *hOGG1* gene.

### 2.3. Distributions of hOGG1 C1245G Polymorphisms in SLE Patients with or without Lupus Nephritis

As shown in Table 3, the 27 SLE patients who harbored the GG genotype were more susceptible to the development of lupus nephritis compared with the other 58 SLE patients who harbored the CC or CG genotypes (15/27, 55.6% *vs.*18/58, 31.0%, *p* = 0.031). Thus, for further comparisons, the 85 SLE patients were divided into 4 groups according to the clinical presentations of lupus nephritis and *hOGG1* C1245G polymorphism. These include SLE patients without nephritis and with the CC/CG genotypes (Group I), SLE patients without nephritis and with the GG genotype (Group II), SLE patients with nephritis and with the CC/CG genotypes (Group III), and SLE patients with nephritis and with the GG genotype (Group IV).

**Table 3:** Distribution of *hOGG1* gene polymorphisms in SLE patients with or without lupus nephritis.

SLE Patients	*hOGG1* Gene Polymorphisms (Case Number, %)		*p*-Value
	*hOGG1* 1245 CC or CG Genotype (*n* = 58, 100%)	*hOGG1* GG Genotype (*n* = 27, 100%)	
Without nephritis	40 (69.0%)—Group I	12 (44.4%)—Group II	0.031 *
With nephritis	18 (31.0%)—Group III	15 (55.6%)—Group IV	

Group I: SLE patients without nephritis and harboring the CC or CG genotypes; Group II: SLE patients without nephritis and harboring the GG genotype; Group III; SLE patients with nephritis and harboring the CC or CG genotype; Group IV: SLE patients with nephritis and harboring the GG genotype; * comparison was made between those harboring CC/CG genotype (Group I + III, *n* = 58) and those harboring GG genotype (Group II + IV, *n* = 27).

### 2.4. Plasma Levels of 8-OHdG in SLE Patients as Related to the Clinical Presentations of Lupus Nephritis and hOGG1 C1245G Polymorphism

**Figure 3:**
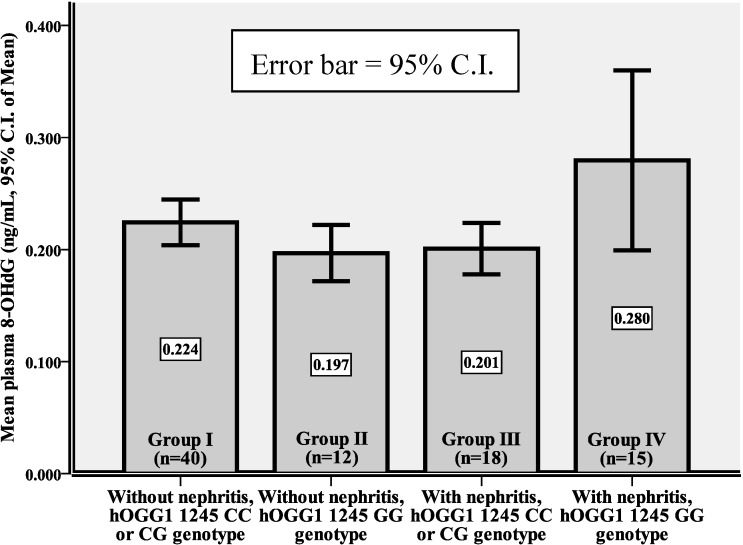
The plasma 8-OHdG in SLE patients with or without nephritis, as related to different *hOGG1* C1245G genotypes (mean levels and 95% C.I.).

In the patients who harbored the CC or CG genotypes (Group I and Group III), there was no significant difference in their plasma levels of 8-OHdG (0.224 ± 0.064 *vs.* 0.201 ± 0.046 ng/mL, *p* = 0.165). In those who harbored the GG genotype (Group II and Group IV), Group IV had higher plasma levels of 8-OHdG than Group II (0.280 ± 0.145 *vs.* 0.197 ± 0.040 ng/mL, *p* = 0.050). In the 33 SLE patients with nephritis (Group III and Group IV), Group IV had higher plasma levels of 8-OHdG than did Group III (0.280 ± 0.145 *vs.* 0.201 ± 0.046 ng/mL, *p* = 0.037). However, such a difference was not observed between Group I and II in the 52 SLE patients without nephritis (0.197 ± 0.040 *vs.* 0.224 ± 0.064 ng/mL, *p* = 0.164). Furthermore, SLE patients with nephritis who harbored the GG genotype (Group IV) had higher plasma levels of 8-OHdG than SLE patients without nephritis who harbored the CC or CG genotypes (Group I) (0.280 ± 0.145 *vs.* 0.224 ± 0.064 ng/mL, *p* = 0.054). Group IV SLE patients with nephritis and with the GG genotype had higher levels of plasma 8-OHdG than did patients in the other groups (*p* = 0.020, ANOVA) (Table 4 and Figure 3).

**Table 4:** Plasma levels of 8-OHdG in SLE patients with or without nephritis as related to the *hOGG1* C1245G polymorphisms.

	Plasma 8-OhdG (M ± SD, ng/mL)		Plasma 8-OhdG (M ± SD, ng/mL)	*p*-Value
SLE patients without nephritis (*n* = 52)	0.218 ± 0.060	SLE patients with nephritis (*n* = 33)	0.237 ± 0.109	0.313
Group I h*OGG1* 1245 CC or CG genotype (*n* = 40)	0.224 ± 0.064	Group III *hOGG1* 1245 CC or CG genotype (*n* = 18)	0.201 ± 0.046	0.165 *
Group II GG genotype (Cys/Cys *hOGG1*) (*n* = 12)	0.197 ± 0.040	Group IV GG genotype (Cys/Cys *hOGG1*) (*n* = 15)	0.280 ± 0.145	0.050 *
*p*-value	0.164 **		0.037 **	0.054 ***
				0.020 ****

M, mean, SD, standard deviation; * compared between SLE patients without nephritis who harbored the CC or CG genotypes (Group I) and SLE patients with nephritis who harbored the CC or CG genotypes (Group III), and between SLE patients without nephritis who harbored the GG genotype (Group II) and SLE patients with nephritis who harbored the GG genotype (Group IV); ** compared between SLE patients without nephritis who harbored the GG genotype and those harboring the CC or CG genotypes (Group II and Group I), and in SLE patients with nephritis who harbored the GG genotype and those harboring the CC or CG genotype (Group IV and Group III); *** compared between SLE patients with nephritis who harbored the GG genotype and SLE patients without nephritis who harbored the CC or CG genotypes (Group IV and Group I); **** compared among all groups (ANOVA).

It was reported that *hOGG1* 1245 GG genotype is related to the development of several human degenerative diseases, including type 2 diabetes mellitus [11], Huntington’s disease [12], chronic obstructive pulmonary disease [13], chronic renal failure [23] and Graves’ ophthalmopathy [14]. Furthermore, the *hOGG1* 1245 GG genotype is associated with multiple vessel involvement in patients with coronary artery disease [22], and with the progression of IgA nephropathy [23]. However, the role of *hOGG1*C1245G polymorphism in lupus nephritis remains unclear. Interestingly, we demonstrated that in our SLE cohort, the 27 patients harboring the GG genotype had a significantly higher rate (15/27, 55.6%) of nephritis than the other 58 patients harboring the CC or CG genotypes (18/58, 31.0%, *p* = 0.031, Table 3). Several previous studies have shown that the GG genotype is related to the higher 8-OHdG concentration in patients with ESRD [18,19]. Thus, it is important to clarify whether the *hOGG1* 1245 GG genotype in patients with lupus nephritis confers a higher oxidative damage to DNA compared with SLE patients without nephritis. As shown in Table 4 and Figure 3, SLE patients with nephritis and with the GG genotype (Group IV) had a higher plasma level of 8-OHdG than did other groups of SLE patients (*p* = 0.020, ANOVA). Therefore, we suspect that the SLE patients harboring *hOGG1* 1245 GG genotype tend to have a higher risk of developing nephritis and accumulating more oxidative damage to DNA in affected tissues.

## 3. Experimental Section

### 3.1. Recruitment of SLE Patients and Healthy Controls

With fulfillment of the American College of Rheumatology (ACR) criteria for the classification of SLE [24], we collected a total of 85 SLE patients (male/female = 12/73) with a mean age of 44.6 years from Outpatient Clinic of the Division of Allergy, Immunology and Rheumatology, Taipei Veterans General Hospital. 45 age-matched healthy controls (male/female = 7/38) with a mean age of 42.6 years served as the controls. We included the same cohort, their demographic data and plasma levels of 8-OHdG, which had been reported previously for other research purposes [20,25]. Among the 85 SLE patients, 33 (38.8%) were confirmed to have lupus nephritis according to the ACR classification criteria [24,25]. Approval from the Institutional Review Board of Taipei Veterans General Hospital was obtained to conduct this study.

### 3.2. Blood Sample Collection, Plasma Separation and Leukocyte DNA Preparation

About 10 mL of venous blood was drawn in a specific tube (VACUETTE^®^, Greiner Bio-one, Monroe, NC, USA) that contained EDTA. After centrifugation at 3000× *g* for 10 min at 4 °C, the plasma and leukocyte-enriched buffy coat were collected separately. The plasma was then subject to the determination of 8-OHdG. After addition with the erythrocyte (RBC) lysis buffer (Bioman, Taiwan), leukocyte DNA was extracted by standard phenol-chloroform procedures and kept at −20 °C until use [26].

### 3.3. Determination of 8-OHdG in Plasma by ELISA

Plasma 8-OHdG was detected by using ELISA kit (High Sensitive 8-OHdG Check ELISA, Japan Institute for the Control of Aging, Nikken SEIL Co., Ltd., Fukuroi, Shizuoka, Japan) according to the manufacturer’s instruction [20,27]. Each reaction was done in duplicate and the mean value was used for data presentation.

### 3.4. Analysis of hOGG1 C1245G Polymorphisms

The *hOGG1* C1245G polymorphisms were detected by direct genome sequencing. Briefly, 1 μL (10 ng/μL) of the leukocyte DNA was amplified in a 50 μL PCR reaction that containing 25 μL of RBC SensiZyme^®^ Hotstart Taq Premix (RBC Bioscience, Taipei, Taiwan), 22 μL of PCR grade H_2_O, 1 μL of each primer (F: 5'-ACTGTCACTAGTCTCACCAG-3' and R: 5'-GGAAGGTGCTTGGGGAAT-3') to generate a 200-bp product [16]. PCR conditions were set as: 95 °C for 10 min followed by 40 cycles of amplification at 95 °C for 30 s, 58 °C for 30 s and 72 °C for 60 s, and a final extension at 72 °C for 7 min. After gel electrophoresis to confirm the DNA band of interest, 30 μL of the PCR-amplified product was subjected to direct sequencing (MB Mission Biotech, Taipei, Taiwan). During sequencing, single C peak at bp1245 indicates a homozygous CC genotype, single G peak indicates a homozygous GG genotype, and concurrent C plus G peaks indicates the heterozygous CG genotype [16,21].

### 3.5 Statistical Analysis

The plasma 8-OHdG concentrations are presented as the mean ± standard deviation (M ± SD). The continuous variables between two groups or among three or more groups were compared using the Student’s *t*-test, Mann-Whitney *U*-test, ANOVA (analysis of variance) or Kruskal–Wallis test when appropriate. A difference was considered significant when a *p*-value was less than 0.05.

## 4. Conclusions

In conclusion, we have provided evidence that *hOGG1* C1245G polymorphism might be one of the multiple factors that confer a higher susceptibility to lupus nephritis and modulate the plasma level of 8-OHdG in patients with SLE.
